# Taking Advantage of Phosphate Functionalized Waterborne Acrylic Binders to Get Rid of Inhibitors in Direct-to-Metal Paints

**DOI:** 10.3390/polym14020316

**Published:** 2022-01-13

**Authors:** Stefano Chimenti, Marco Cerra, Tito Zanetta, Jose Ramon Leiza, María Paulis

**Affiliations:** 1 POLYMAT, Kimika Aplikatua Saila, Kimika Fakultatea, University of the Basque Country UPV/EHU, Joxe Mari Korta Zentroa, Tolosa Hiribidea 72, 20018 Donostia-San Sebastián, Spain; stefano.chimenti@polymat.eu; 2 Vinavil SPA, Via Toce, 7, 28844 Villadossola, Italy; m.cerra@vinavil.it (M.C.); t.zanetta@vinavil.it (T.Z.)

**Keywords:** waterborne phosphate-stabilized binder, corrosion protection, inhibitor-free, direct-to-metal paint, paint formulation

## Abstract

In this paper, two phosphate functionalized acrylic binders are formulated to yield direct-to-metal paints without using corrosion inhibitors. The difference between both binders is the addition of polystearylacrylate crystalline nanodomains in one of them, and an amorphous methyl methacylate-co-butyl acrylate copolymer in the other. The water sensitivity, mechanical stability, adhesion, and the performance of the paints against corrosion (high humidity resistance, accelerated weathering, and salt-spray tests) are assessed and compared with a DTM paint formulated from a commercial binder. The performance of both phosphate functionalized paints formulated without corrosion inhibitors in high humidity and weathering tests is superior to the commercial DTM paint formulated without corrosion inhibitors and similar to the DTM paint formulated with them. Furthermore, the paint based on the amorphous copolymer binder provides significantly good performance in the salt spray test (even superior to that of the DTM paint formulated with corrosion inhibitors).

## 1. Introduction

Solventborne polymeric binders have been traditionally used in paint formulations for metal surfaces. However, despite solventborne paints are well known to provide high barrier protection and high gloss finish [1,2,3], they require long drying times and they produce high emission of volatile organic compounds (VOCs), which makes them less environmentally friendly [4,5]. Due to this, and due to the more stringent government regulations about the VOC emissions [4,5], waterborne polymer dispersions for anticorrosive paints are gaining relevance for the protection of steel surfaces exposed to the atmosphere. 

In a waterborne paint formulation, the binder accounts for about 50% of the total weight, while the remaining part of the paint comprises inorganic pigments and other agents such as defoamers, dispersants, thickeners, wetting and coalescing agents and anticorrosion inhibitors. Typically, the inorganic pigments have several roles such as providing color (color pigments such as white (TiO_2_, ZnO [6]), red (Fe_2_O_3_ [7]) or blue (CoAl_2_O_4_ [7,8,9]), reducing the cost (extender pigments, such as coarse CaCO_3_) and providing protection against corrosion (Zn_3_(PO_4_)_2_, Ce(NO_3_)_3_ and La(NO_3_)_3_ [10]). Anyway, the polymeric binder plays a key role in the paint formulation as the properties of the resulting coating depend in a large extent on it. Therefore, its correct choice and design is of a paramount importance. 

One possibility to improve the corrosion protection properties of the binders used in anticorrosion applications is to increase the barrier properties of the polymer. Recent attempts in this direction include the introduction of semicrystalline nanodomains in the latex [11,12,13], using polymerizable surfactants to avoid migration or formation of hydrophilic pockets in the films [14], producing surfactant-free latexes by MacroRAFT mediated emulsion polymerization [15], and rendering the polymer more hydrophobic to reduce permeation of corrosion agents (oxygen, water and ions) [16,17]. Nevertheless, barrier properties provided by a binder are not able to provide full corrosion protection to a coating, and hence the anticorrosion performance is markedly improved when anticorrosion pigments, inhibitors or additives are included in the paint formulation or in the binder itself. In this sense, recently, waterborne polymer dispersions (acrylic, epoxy, polyurethanes, and hybrids systems) have been modified by including in the dispersions boron nitride nanoparticles/nanosheets [18,19,20,21], graphene oxide [22,23,24], polyaniline nanoparticles [25,26,27], nanoferrite [28], and carbon nanotubes [29] among others. In addition to that, for a long lasting and efficient corrosion protection of the coated material, the metal substrate is usually chemically pretreated to provide enhanced corrosion protection as well as to increase its adhesion to the organic coating. In other words, outstanding corrosion protection is achieved in these cases by the combination of the organic coating and the metal pretreatment layer [30,31,32,33,34,35].

However, in the last two decades the coating industry is moving forward toward the development of single layer direct to metal coatings (DTM) in an attempt to reduce cost while maintaining efficiency and performance [36]. DTM coatings are applied directly to a metal surface with the ability to adhere to it without the need for extensive cleaning or pretreatment layers. In fact, one strategy concerning the use of corrosion inhibitors is to passivate the metal substrate using a passivating moiety in the binder that can interact with the substrate upon application of the DTM coating. Thus, Reyes et al. [37] explored the use of a phosphate-based surfactant (Rhodafac 610) to produce styrene-acrylic polymer latexes and tested the corrosion properties of the films that were found to be dependent on the particle size of the latex. The corrosion performance of the films was modest (EIS impedance values at 0.1 Hz decreased from 5 × 10^5^ to 1 × 10^4^ Ω·cm^−2^ after 21 days in a 3 wt% NaCl solution). In the same vein, Breucker et al. [38] reported a route to produce phosphorus-functionalized polyurethane dispersions with improved adhesion to the metal. Although the dispersions were film forming and potentially interesting for anticorrosion coatings, this was not demonstrated. Lin et al. showed that by blending a phosphatizing agent with a waterborne polymer dispersion it was possible to produce a phosphatization layer on the metal surface during the application of the coating, substantially enhancing the protection of the metal [39,40].

In a recent work [41], we reported the synthesis of a waterborne (meth)acrylic binder with phosphate functionality (by using a phosphate reactive surfactant, SIPOMER PAM 200). Films made from this binder presented excellent corrosion resistance when applied on steel panels under controlled conditions of temperature and relative humidity (e.g., 23 °C, RH = 60%). The phosphate groups present on the surface of each polymeric particle were able to interact with the hydroxyl groups on the surface of the steel and to produce a passive iron phosphate layer covalently bonded to the polymeric film. Therefore, the combination of substrate passivation and good water resistance of the polymeric film produced excellent corrosion protection for extended period under harsh corrosive conditions (exposure to a 5 wt% NaCl salt spray) [41]. Afterwards, the anticorrosion performance of the phosphate containing latex binder was further enhanced by improving the barrier properties of the bare film through the introduction of crystalline nanodomains in the polymer particles (crystalline polystearyl acrylate (PSA) nanodomains) [13]. Nevertheless, all of these trials were made coating the metal substrates with the binder alone, before including it in a final paint formulation.

Herein, novel waterborne polymer dispersions containing phosphate functionalities have been assessed in a standard DTM paint formulation without the use of any anticorrosion pigment. A common white paint formulation was used, which was not optimized for the novel binders used but which can help to envisage the potential of the binders produced in DTM paints resistant to corrosion. The corrosion protection of the novel waterborne phosphate stabilized binders-based paint systems have been tested and then compared with a DTM paint formulated using a commercial anticorrosion binder with corrosion inhibitors and without them.

## 2. Experimental

### 2.1. Materials

Methyl methacrylate (MMA, Arkema, Colombes, France), n-butyl acrylate (BA, BASF, Ludwigshafen, Germany) and stearyl acrylate (SA, BASF) were used without further purification. Dodecyl diphenyl oxide disulfonate (Dowfax 2A1 45%, Dow Chemical Company, Midland, MI, USA) was used as an anionic emulsifier. Sipomer^®^ PAM200, a phosphate ester of polypropylene glycol monomethacrylate (Solvay, Brussels, Belgium), was used as a surfmer. Azobisisobutyronitrile (AIBN, Sigma-Aldrich, Saint Louis, MO, USA) and potassium persulfate (KPS, Peroxitalia, Fornovo, Italy) radical initiators were used as received. Sodium bicarbonate (NaHCO_3_, BRENNTAG, Essen, Germany) was used as a buffer, to reduce the electrostatic interaction among droplets and to control the viscosity of the miniemulsion. Tertbutyl hydroperoxide (TBHP, Peroxitalia) and Bruggolite FF6 (Brüggemann, Heilbronn, Germany) were used as redox couple. Deionized water was used as a solvent in all reactions performed. For the paint formulation the following ingredients were used; Tego Airex 902W (Evonik, Essen, Germany) was used as deaerator, Disperbyk 191 (BYK, Wesel, Germany) as dispersant agent, R706 TiO_2_, ZnO (Chemours, Wilmington, DE, USA) and CHB2 talc were used as pigments and fillers respectively. Zinc phosphate Z-plex 111 (Halox, Hammond, IN, USA) was used as corrosion inhibitor, butyl carbitol as coalescing agent, Tego Foamex 1488 (Evonik) as defoamer and Acticide MBS (Thor, Casale Litta, Italy) biocide were included also. Lastly, AMP 95 (Angus, Buffalo Grove, IL, USA) was used as alkalizing agent, Byk 3455 (BYK) as wetting agent and Tafigel PUR 44 (Münzing, Abstatt, Germany) as thickener. UniClean 251 (Atotech, Birmingham, UK) or Acetone (Sigma-Aldrich, Saint Louis, MO, USA) were used as degreasing agents for the steel substrates. HCl 1M solution (Aldrich) was used in the cleaning treatment of the steel substrates. High purity NaCl (Corrosalt, Ascott-Analytical, Tamworth, UK) was used for the preparation of a 5 wt% solutions during the corrosion tests.

### 2.2. Synthesis of the Phosphate Stabilized Binders

The details of the synthetic procedure to produce the phosphate stabilized binders is described elsewhere [13,41]. Here for brevity, a short description is included below, while a more detailed description has been included in the Supplementary Materials section. The latexes containing phosphate functionalities were synthesized by seeded semibatch emulsion polymerization [41] and the synthesis was carried out in the R and D department of Vinavil (Italy).

#### 2.2.1. Poly(MMA-co-BA) Latex

A copolymer based on MMA/BA with a composition of 50/50 wt% and with a solids content of 50% was prepared from a seed of same composition and solids content of 13%. The reaction was carried out in a glass jacketed reactor under a N_2_ atmosphere at 70 °C in semibatch conditions by feeding a mixture of the monomer (MMA/BA = 50/50), water, and 2 wbm % of Sipomer PAM200 (SIP) during 4 h. Upon finishing the monomer pre-emulsion feeding, a redox initiator couple (5 wt% Bruggolite (FF6) solution and 5 wt% tertbutyl hydroperoxide (TBHP) solution) was fed at the end of the reaction to reduce the concentration of the unreacted monomers at a flow rate of 0.733 g/min for 30 min. The resulting latex was labelled MB. The recipes to produce the seed and the final MB latex are reported in the Supplementary Materials section.

#### 2.2.2. Poly(SA/MMA-co-BA) Latex

Core-shell latexes (PSA core and poly(MMA-co-BA) shell) were synthesized by seeded semibatch emulsion polymerization following the polymerization strategy proposed by in previous works [11,12,13]. The seed, based on PSA, was prepared by a batch miniemulsion process in which the whole amount of SA was left to polymerize in situ during 3 h; the seed latexes were labelled SA40s and SA50s, respectively. Then, a preemulsion based on the shell monomer mixture (MMA/BA), water and 2 wbm % of Sipomer PAM200 (SIP) was fed to the seed latexes. After the end of the preemulsion feeding, the reaction was post-polymerized for an additional hour and a redox initiator couple was fed at the end of the reaction in order to reduce the concentration of the unreacted monomers and to reach a final solids content of 45%. Namely, 5 wt% Bruggolite (FF6) solution and 5 wt% tertbutyl hydroperoxide (TBHP) solution were fed separately at 0.733 g/min for 30 min. The difference between SA40 and SA50 latexes was the SA comonomer amount in the final polymer composition, namely 40 and 50 wbm %, respectively. 

### 2.3. Preparation of Waterborne Paints

The waterborne paints were prepared by using a standard formulation for DTM paints in a one step process (the formulation is listed in Table 1). In the Supplementary Material the main properties of each ingredient are briefly described. First of all, water, defoamer (TEGO AIREX 902 W, Evonik), dispersant (DISPERBYK 191, BYK) and a low amount of the binder (5.9 wt% with respect of the total paint weight) were premixed at 900 rpm using a high-speed disperser blade (DISPERMAT CN10). The pigment (TiO_2_ Type R706, Chemours) was then slowly added and stirred at 2000 rpm. After 10 min the stirring rate was reduced to 900 rpm and the filler (Talc CHB2), coalescing agent (butyl carbitol) and the rest of the binder were incorporated. At the end, defoamer (Foamex 1488, Evonik), biocide (Acticide MBS, Thor), alkalizing agent (AMP 95, Angus), wetting agent (Byk 3455), and a thickener (Tafigel PUR44, Münzing) were added and the paint was let under stirring at 900 rpm for 10 min. The resulting paints were labelled P_MB, P_SA40, P_SA50. For comparison purpose, a commercial binder (CB) was also formulated with and without the addition of corrosion inhibitors, labelled CB_1 and CB_0 respectively. Zinc phosphate (Z-plex 111, Halox) and an organic zinc complex (Naziln FA179, Elementis, London, UK) were incorporated in CB_1 paint as corrosion inhibitors. 

### 2.4. Substrate Cleaning and Paint Application

The anticorrosion performance of the prepared paints was tested on steel substrates; Q-LAB type bare steel smooth mill finish (QD-612). Two substrate cleaning procedures were considered in this work. On the one hand, a simple cleaning with acetone was adopted, and labeled as method A, in which any contamination by wax or grease of the steel substrates was removed simply by rinsing with acetone. On the other hand, the steel substrates were first degreased with Uniclean 251 solution at 343 K in a shaking bath for 5 min followed by 1 min of pickling in HCl solution (1:1). After each step, the steel substrate was carefully rinsed with distilled water and at the end of the cleaning process, it was dried with compressed air. This cleaning process was named U.

After the chosen cleaning process, the paints were uniformly applied on the steel substrates with a roll bar applicator aiming to obtain a dry film of 100 µm. Films made from P_MB paints were dried at 296 K and 60% of relative humidity (RH), whereas films based on P_SA paints were dried either at 296 K and 60% RH or at 333 K and 30% RH. These drying conditions have been proved with the bare binders to be optimal for the formation of a phosphate passive layer that provides corrosion protection to the metal substrate upon the coating film formation [13,41].

### 2.5. Characterization and Testing Methods

Water sensitivity was assessed by liquid water uptake (WU) test, that consists in monitoring for 14 days the weight gain of circular polymeric specimens (diameter = 24 mm, thickness = 2.3 mm) immersed either in water or in a 3.5 wt% NaCl aqueous solution.

Mechanical stability was assessed by loading the latex in the stability tester and stirring at 10,000 ± 200 rpm by means of an impeller. During the test, foaming and progressive flocculation are monitored visually. After 10 min of stirring, the amount of coagulum (in ppm) is quantified by filtering the latex with a 325 mesh (44 μm) steel filters and it is compared with the values of coagulum before the mechanical test. Coagulum amounts below 100 ppm are acceptable, and the latex is considered valid for the subsequent formulation step. The mechanical stability test is a rapid, simple method of estimating the colloidal stability of the latex by high-speed stirring [42]. The evaluation of mechanical stability after high-speed stirring is important since it represents the latexes processability in the industrial scale. A latex has to be formulated after its production, which means high-speed mixing with other paint’s components and hence it has to be stable under high shear rate conditions. 

Adhesion of the dry paint on the substrate was assessed by the peel test. The test consists in observing whether the film is peeled off when a tape attached to it is removed. In detail, a cross-hatched pattern is cut into the coating, a tape (Tesa 4104) is applied and removed, and the coating removal is assessed against the established rating scale described in the ASTM D3359 test method B [43]. Generally, the dry-thickness of the film applied according to the method B has to be lower than 125 µm.

In order to evaluate the performance of the paints against corrosion, high humidity resistance, weathering, and salt spray tests were carried out. According to the ISO 6270-2:2005 [44], high humidity resistance test consists in evaluating the water resistance, and hence corrosion, of coated specimens in an atmosphere maintained at 99% RH and 313 K, so that condensation forms on the specimens. In detail, steel panels were coated with the different paints, then scribed with a knife (6 cm long scribe) and maintained at the testing conditions for 250 h. At the end of the test, the presence of rust, blistering, loss of adhesion or embrittlement were evaluated. 

In the weathering test scribed panels were exposed to 200 h periods of high humidity followed by 200 h periods of light/condensation exposure (4 h UV light exposure to a Xenon lamp, which simulates the short-wave solar radiation at 340 nm at 60 °C, alternating with 4 h condensation at 50 °C) for a total of 2000 h. For the weathering component of this test, a standard ultraviolet light condensation cabinet, according to the ASTM G53 standard [45], was employed. Finally, accelerated neutral salt spray tests (NSS) were carried out in a Q-FOG CHR 600 HSC (Q-LAB) equipment, following the specification described in the standard test ASTM B117. 

Scanning electron microscopy assisted with energy disperse X-ray analysis (SEM-EDX) was used to visualize the morphology of the coatings and to analyze the interface between the coating and the substrate in terms of its chemical composition (Fe and O). It is worth mentioning that phosphorous could not be detected due to the too low concentration present in the final paint composition. Previously, the coated steel samples subjected to the high humidity tests were cross sectioned. All of the measurements were performed using a bench top SEM 3030 Hitachi (Tokyo, Japan) operating at 8 kV. Furthermore, the elemental composition of the coating applied onto the steel substrate was obtained using a Quantax EDS Bruker. 

## 3. Results and Discussions

### 3.1. Waterborne Binders Containing Phosphates Functionalities

Coagulum free poly(MMA-co-BA) (MB) and poly(SA/MMA-co-BA) latexes (SA40 and SA50) with 50% and 45% solids respectively, were successfully synthesized by seeded semibatch emulsion polymerization carried out under starved conditions as reported elsewhere [13,41]. The average particle sizes, the thermal properties, and the morphological characterization of the synthesized latexes (MB, SA40, and SA50) are also included in the Supplementary Material. Within all of the properties that a polymeric binder should provide to the formulated paint with suitable anticorrosion performance, the water resistance is one of the most important. Water uptake test is an easy way to quantify the extent of the water retained inside a polymeric film and hence its water resistance. Generally, this test is performed in distilled water aiming to evaluate the effect of mere water without considering any electrolytes. Nevertheless, in the present work, the water uptake test was carried out in both distilled water and in a 3.5 wt% NaCl solution in order to evaluate the water resistance of the film in testing conditions closer to the marine environment and hence more aggressive from the corrosion point of view. Table 2 reports the water uptake values of the films collected after 14 days of immersion either in distilled water or in 3.5 wt% NaCl solution. For comparison purposes, the liquid water uptake of film cast from the commercial benchmark binder (CB) is also reported.

The water uptake behavior of the tested films was the same observed in the previous works [13,41]. Particularly, it can be noticed that, drying the films based on SA latexes at 60 °C is beneficial for the water resistance as confirmed by a drop in the water uptake either in distilled water or in the salt solution. This reduction is mostly related to a better film formation at temperatures above the melting point of the crystalline nanodomains (Tm _Crystalline PSA_ = 50 °C) [11,12,13]. On the contrary, the drying temperature did not affect the water resistance of films made from either MB or CB and hence only the values for films dried at 23 °C are reported. Moreover, the films cast from SA40_T60, SA50_T60, and MB showed lower water uptake (either in distilled water or in 3.5 wt% NaCl solution) than the films prepared from the commercial latex. This confirms the beneficial effect of using a phosphate surfmer (the use of polymerizable surfactant is known to reduce the water sensitivity of the polymeric films [14,46,47]) and of the crystalline nanodomains homogeneously dispersed in the film prepared from SA based latexes (see the Supplementary Materials section for the TEM characterization of the SA40 and SA50 latex particles and of the films cast from them). 

However, it is worth mentioning that the water uptake measured in 3.5 wt% NaCl solution were lower than those obtained in distilled water. This result might be related to a reverse osmosis. Generally, when the water permeates into a film made from a latex, it dissolves the internal water-soluble substances, such as the surfactant molecules, producing electrolytes. Hence, due to osmosis, more water is driven into the film. On the other hand, in a NaCl solution the electrolyte concentration in the solution results to be higher than the electrolyte concentration inside the film after water permeation and hence the total water uptake of the film is reduced due to the reverse osmosis [48,49,50]. 

Before formulating the DTM paint, the mechanical stability of the synthesized binders was tested. As it can be seen in Table 3, when the latex pH was increased from 7 to 8 by the addition of ammonia solution, the amount of filtered coagulum at 325 mesh was substantially reduced and it remained under the permitted limit of 100 ppm. The addition of a base increases the colloidal stability [51]. Therefore, the pH of all of the formulated waterborne latexes was corrected to 8. 

### 3.2. Performance of DTM Paints

It is well known that the basic features in order to design an efficient waterborne paint for corrosion protection are adhesion, barrier protection and flash rust resistance; for this reason, the assessment of these properties are presented below. Namely, adhesion resistance, flash rust resistance, high humidity, and weathering tests were performed for all of the paint formulations. Table 4 presents the adhesion test results for paints based on MB, SA40 and SA50 latexes dried at 296 and 333 K (P_SA40 and P_SA50 dried at 296 K and P_SA40_T60 and P_SA50_T60 dried at 333 K) and CB (CB_0 and CB_1), applied onto steel substrate, cleaned with the previously mentioned procedures (A and U).

**Figure 1:**
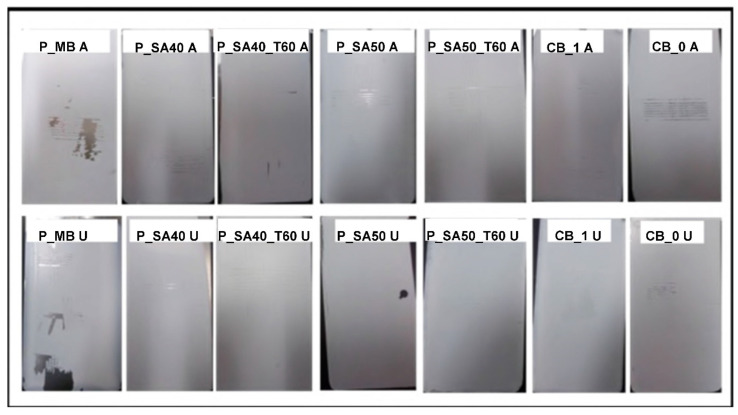
Results of adhesion test of the formulated paints onto steel substrates.

At first glance, it can be noticed that P_MB paints showed the lowest adhesion properties in comparison with the other specimens tested. The coatings have flaked along the edges of the cut in large ribbons, and whole squares have detached (Figure 1). However, if we take a closer look at P_MB A and P_MB U specimens, the majority of the coating peeled off is at the boundaries of the cross cut while the central part resulted less damaged. This finding may suggest the presence of localized voids or defects that affect the interfacial bonding between coating and substrate and hence the fracture, generated during the tape peeling off, can propagate along the weakest point [52]. All of the remaining coatings showed good to excellent adhesion properties. Moreover, in the case of P_SA films dried at 333 K and applied on substrate cleaned with procedure U, none of the square of the cross-cut was detached after the peeling (Figure 1), which means that the higher drying temperature reduces the concentration of voids and defects [13] and hence the fracture formation and propagation after the tape peeling. Since the only difference between P_MB with P_SA paints is the presence of semicrystalline nanodomains in the latter, it can be concluded that these crystalline domains provide cohesion to strengthen the amorphous phase. 

**Figure 2:**
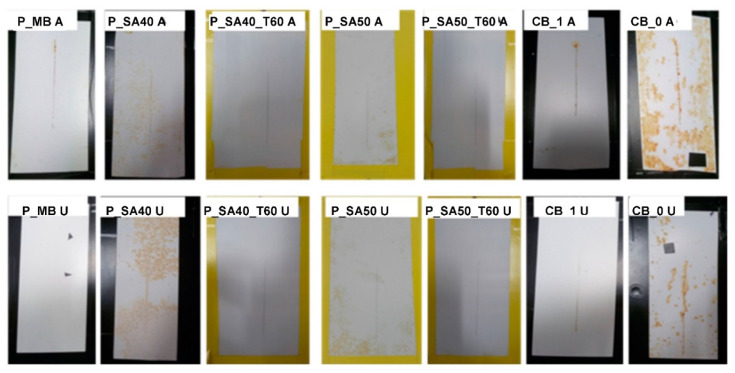
Results of high humidity test for scribed specimens coated with the formulated paints on steel cleaned with the different cleaning procedures (A and U) after 250 h of exposure to RH = 99% and T = 40 °C.

The beneficial effect of using waterborne latexes containing phosphate functionalities showed up in the high humidity resistance test. In fact, as it can be seen in Figure 2, coatings cast from P_MB, P_SA40_T60 and P_SA50_T60 paints were able to protect the steel substrate without the addition of any corrosion inhibitors, whereas substrate protection by paints based on the commercial binders was only achieved for the formulation containing inhibitors (CB_1).

Either CB_0 A and CB_0 U clearly presented signs of corrosion and, in both cases, the rust started to form in the whole specimen and not only in the scribe. In the case of films cast from P_SA paints and dried at 296 K (P_SA40 A, P_SA40 U, P_SA50 A and P_SA50 U), corrosion started from the scribe and then propagated in the whole surface of the specimen. These results agree with the obtained water uptake values and reflect what has been seen in a previous work [13] where drying of the SA based films at 296 K or 333 K affected the final barrier properties and hence the anticorrosion performance. Another important feature provided by phosphate containing waterborne latexes resulted in the ability to avoid delamination of the coating as a result of the formation of corrosion products. In fact, even if P_MB, P_SA40_T60 and P_SA50_T60 presented some corrosion spot localized at the incision (where the steel surface is directly in contact with oxygen and moisture), the corrosion did not propagate to the near coating-substrate interface, which can be considered as an indirect proof of the formation of an iron phosphate layer at the interface (as demonstrated for the bare films that remained tightly attached to both the coating and the steel surface [13,41]). 

**Figure 3:**
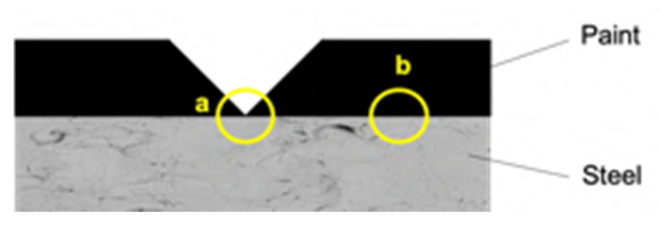
Scheme of the cross section of the coated specimens. Note: SEM-EDX analysis was carried out at (**a**) the scribe and at (**b**) non-scribed region.

For comparison purpose, the cross-section of P_MB U, P_SA40_T60 U and CB_0 U specimens has been investigated by scanning electron microscopy assisted with energy dispersive X-ray spectroscopy (SEM-EDX). Namely, as it shown in scheme in Figure 3, the scribe and a non-scribed region of the cross section have been analyzed. 

**Figure 4:**
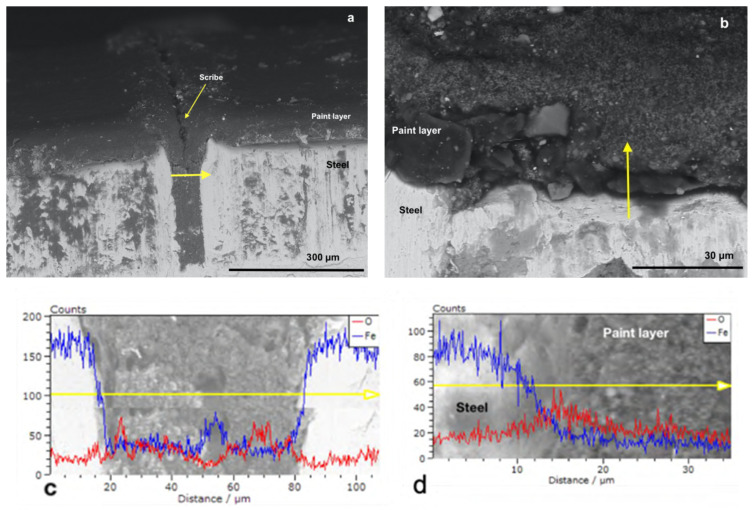
SEM micrographs with EDX linescan (**c**,**d**) of the cross-section of P_MB U coated specimens at (**a**) the scribe and at (**b**) no-scribed region. Red and blue spectra correspond to O and Fe, respectively.

**Figure 5:**
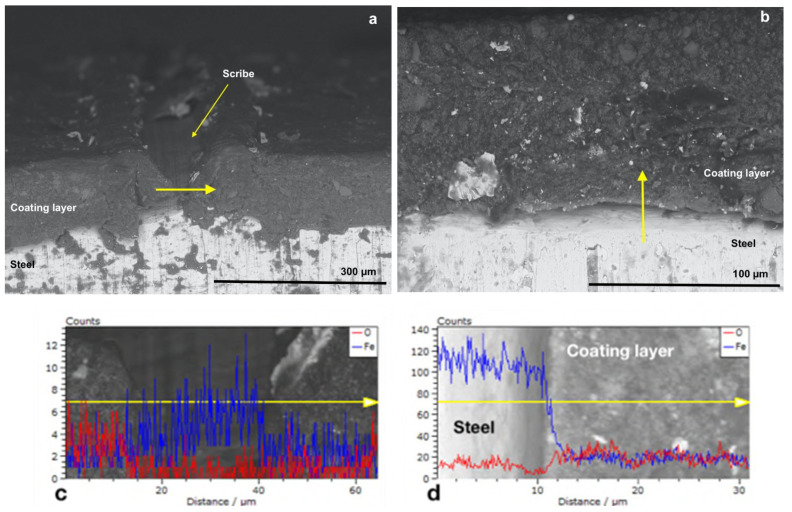
SEM micrographs with EDX linescan (**c**,**d**) of the cross-section of P_SA40_T60 U coated specimens at (**a**) the scribe and at (**b**) no-scribed region. Red and blue spectra correspond to O and Fe, respectively.

Figure 4b and Figure 5b show the cross-sectioned specimens (P_MB U and P_SA40_T60 U) at the non-scribed region with the tracks in which the EDX linescan analysis were performed marked by a yellow arrow. EDX linescan analysis were carried out since it is a powerful technique that allows to detect qualitatively the presence of corrosion products, along the selected track, by monitoring the iron and oxygen counts profiles (counts is referred to the number of X-ray photons emitted and hence the intensity of the emitted X-ray radiation [53]). Note that although Phosphorous was also targeted, due to the low concentration of the polymerizable surfactant present in the overall formulation, it was not detected above the noise. Figure 4d and Figure 5d present the profiles of iron (in blue) and of oxygen (in red) recorded in the direction of the yellow arrow, from the steel to the paint at the non-scribed region of the cross-section (P_MB U and P_SA40_T60 U). The steel phase appears bright in the micrographs and with higher counts of iron, with respect to the one of oxygen, indicating the absence of iron oxides (in the case of the presence of iron oxides the oxygen profiles should have presented some peaks with counts closer to the ones of iron). Moreover, moving along the linescan track, from the steel to the coating layer, the drop in the Fe count profile indicates the absence of Iron in the latter and hence the absence of corrosion products. 

On the other hand, as expected, some rust was found at the scribe (Figure 4a and Figure 5a) as confirmed in the EDX linescan profiles (Figure 4c and Figure 5c). The presence of peaks (higher counts) in the profiles of either Oxygen (approximatively at the linescan track length of 25 and 70 μm in Figure 4c and at 5–10 μm in Figure 5c) or Iron (approximatively at the linescan track length of 50 μm in Figure 4c and 30 and 35 μm in Figure 5c) in the scribe indicates that some corrosion products developed during the corrosion test. However, it is noteworthy the fact that the corrosion did not propagate in the coating and remained confined in the scribe. Indeed, in the nearby region of the scribe the lower count of oxygen profile with respect to the count profile of Iron indicates the absence of rust (Figure 4 and Figure 5).

**Figure 6:**
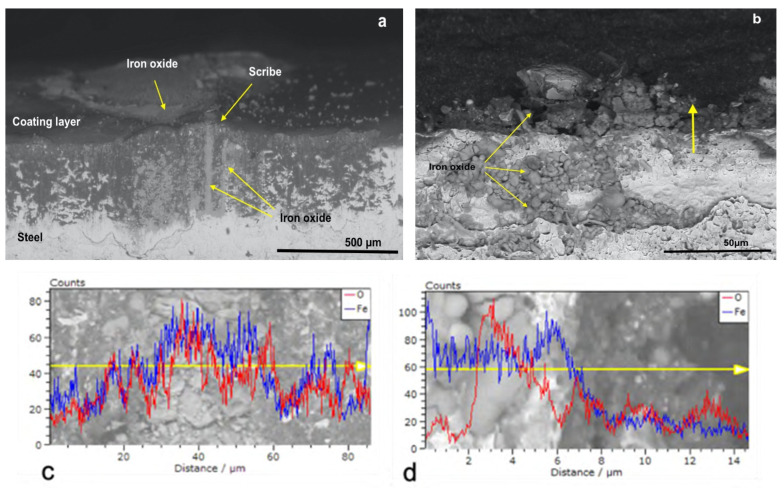
SEM micrographs with EDX linescan (**c**,**d**) of the cross-section of CB_0 U coated specimens at (**a**) the scribe and at (**b**) no-scribed region. Red and blue spectra correspond to O and Fe, respectively.

Figure 6a shows the micrograph of CB_0 U at the scribe but, in this case, the scribe was not well defined as in the case of P_MB U and P_SA40_T60 U (Figure 4a and Figure 5a, respectively). The EDX analysis of the scribe (Figure 6c) revealed the presence of several peaks in both the Oxygen and Iron count profiles, which indicated that, during the corrosion test, the formation of iron oxides occurred in larger extent, filling the scribe. Moreover, it seems that the corrosion propagates to the nearby region as confirmed in the EDX linescan analysis (Figure 6c, peaks in both the Oxygen and Iron count profiles are present). Additional proof of the poor protection of CB_0 U is given by the micrograph of the cross section taken in the non-scribed region and its EDX analysis (Figure 6b,d). The presence of corrosion products in the steel phase beneath the paint layer (grains-shape iron oxides that appear darker than the steel phase in Figure 6b) indicated that, despite the integrity of the coating system (non-scribe region analyzed in this case), the water molecules were able to diffuse to the underlying metal surface and to trigger the corrosion that propagated in both the steel and the coating phase. The presence of several peaks in the count profile (EDX line scan in Figure 6d) of both oxygen and iron either in the steel phase or in the coating phase agreed with the previous observation and confirm the propagation of the corrosion.

**Figure 7:**
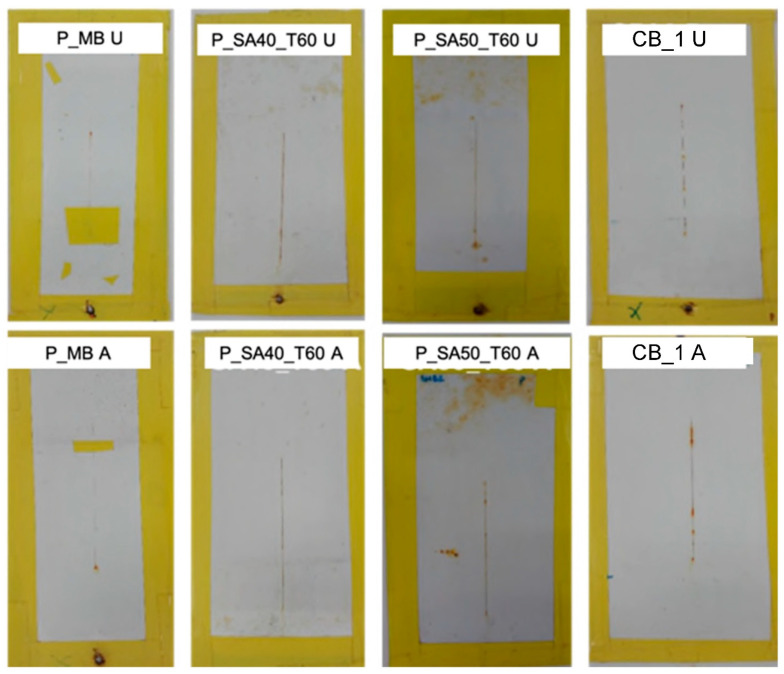
Results of cyclic high humidity-weathering test for scribed specimens coated with the formulated paints on steel cleaned with the different cleaning procedure (A and U) after 2000 h of exposure. Note: P_SA40 and P_SA50 coated specimen and dried at ambient temperature have not been tested due to the degradation of those after the first cycle of exposition at high humidity.

The high humidity resistance test is referred to constant climate conditions. However, it can be combined with repetitive cyclic variations in temperature, humidity or even radiation. For instance, irradiation with UV is primarily used to assess the photochemical resistance of the binder system. However, it may also give a good account on the overall resistance of the entire coating against weather, when combined with humidity [44,45]. A weathering cycling test substantially attempts, more closely than the high humidity test, to simulate the outdoor conditions (non-marine) in a laboratory environment (closely controlling levels of humidity, UV level, and temperature) and it is recommended for paint coatings being used in typical (non-marine) atmospheric conditions exposure. As it can be seen in Figure 7, MB and SA40_T60 and SA50_T60 based paints (substrate cleaned with procedure A or U) performed similarly to the commercial DTM benchmark CB_1 (with corrosion inhibitors in the formulation), but without the addition of any corrosion inhibitors. Notably, the performance of paints based on the binders prepared in this work was similar to the commercial one, which indicates that they were able to provide at least the same protection coverage under cyclic and prolonged corrosion conditions similar to the outdoor exposure. 

**Figure 8:**
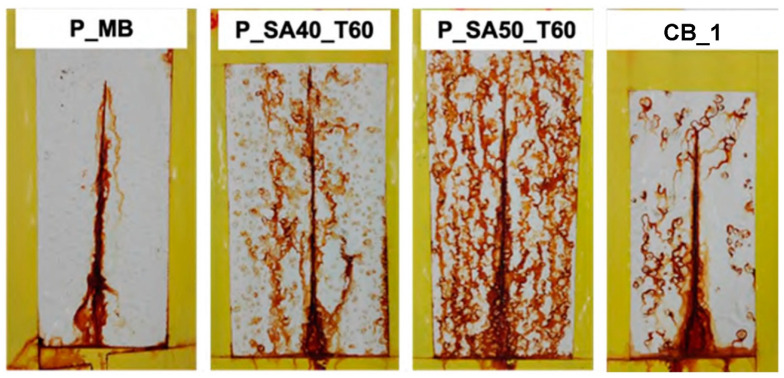
Results of salt spray test for scribed specimens coated with the formulated paints after 240 h of exposure to 5 wt% NaCl salty fog.

An accelerated corrosion test (i.e., salt spray test) was also carried out, and Figure 8 shows the result of coated steel panels after 240 h of exposure to the 5 wt% NaCl fog. Since the cleaning procedure of the steel surface did not show particular difference in the previous paint tests, just the procedure A was carried out.

At first glance, the P_MB coat showed good corrosion protection if compared with the commercial one (CB_1). As it can be noticed, the corrosion occurred at the scribe, but did no propagate whereas in CB_1 signs of corrosion appeared in the whole coating surface area. This result provides further evidences of the anticorrosion protection without the addition of anticorrosion inhibitors provided by MB based paint.

On the contrary, P_SA coats dried at 60 °C showed poor performance in salt spray as coated specimens underwent severe corrosion after 240 h of exposure. This result was not expected especially since the bare binders’ films presented a better performance (+800 h in salt spray test, compared to 400 h for MB binder; see the Supplementary Materials section) and lower salty water uptake than the rest of the binders. Admittedly, we do not have an explanation for this inconsistency in the salt-spray test of the binders and the paints formulated from these. However, it is worth recalling, as discussed in the introduction, that formulating a paint is a complex and sophisticated technique that relies in trial-and-error. Furthermore, it is known that small changes in the binder composition might require substantial adjustments in the formulation of the optimal paint. 

## 4. Conclusions

The incorporation of phosphated waterborne latexes (MB, SA40, and SA50) into commercial paint formulations for direct to metal (DTM) application was addressed in this work and compared with a DTM paint formulated with a commercial binder. The presence of phosphate functionalities, that showed in the previous works the ability to enhance the anticorrosion properties of waterborne binders, allowed to design and formulate paints that provide anticorrosion properties comparable with the one provided by the commercial system, but notably without the use of any corrosion inhibitors. In fact, contrary to the commercial binder formulated without the corrosion inhibitors, P_MB, P_SA40_T60, and P_SA50_T60 coatings successfully protected the steel substrate at high humidity conditions, as well as in a weathering test of 2000 h (cyclic exposure to UV radiation and high humidity conditions). The outcome of the corrosion and weathering tests provided an important proof of the powerful outlook of these systems in the corrosion protection, especially since they could reduce the cost of the final coating systems avoiding the addition of corrosion inhibitors. It is worth mentioning that in the case of SA based coating, drying the film at 333 K was critical to provide corrosion protection, which is consistent with the results described in our previous work [13]. In any case, these drying conditions were not enough to provide P_SA coatings with good protection against more aggressive corrosive conditions of the salt-spray, which indicates the necessity of an optimization of the formulation for the binder containing the nanocrystalline domains. However, it is remarkable to point out that P-MB coating provided even better corrosion protection than the commercial DTM paint formulated with corrosion inhibitors, in the demanding salt spray test. As a conclusion, the potential of including surface phosphate functionalities in waterborne polymer particles to get rid of corrosion inhibitors in DTM paint formulations has been demonstrated. 

## 5. Patents

A Method for Providing Coating Systems With Corrosion-Protective Properties. S. Chimenti, J.R. Leiza, M. Paulis, J.M. Vega, E. García. WO2020043327A1.

## Figures and Tables

**Table 1:** Waterborne paint formulations with corrosion inhibitors (CB_1) and without corrosion inhibitors (P_MB, P_SA40, P_SA50, CB_0).

		Without Inhibitors P_MB, P_SA40, P_SA50 and CB_0		With Inhibitors CB1	
Ingredients	Name	wt%	Amount (g)	wt%	Amount (g)
Water		28.2	84.6	20.4	61.2
Defoamer	Tego airex 902W	0.2	0.6	0.2	0.6
Dispersant	Disperbyk 191	0.6	1.8	0.6	1.8
Binder	Different ones *	5	15.0	5	15.0
Pigment	TiO_2_ type R706	15	45.0	15	45.0
Pigment	Zinc oxide	-	-	2.9	8.7
Inhibitor	Z-plex 111	-	-	3.6	10.8
Filler	Talc CHB2	5	15.0	5	15.0
Coalescing agent	Butyl carbitol	2	6.0	2	6.0
Binder	Different ones *	42	126.0	42	126.0
Defoamer	Foamex 1488	0.2	0.6	0.2	0.6
Biocide	Acticide MBS	0.1	0.3	0.1	0.3
Inhibitor	Naziln FA179	-	-	1.3	3.9
Alkalizing agent	AMP95	0.3	0.9	0.3	0.9
Wetting agent	Byk 3455	0.4	1.2	0.4	1.2
Thickener	Tafigel PUR44	1	3.0	1	3.0

* CB commercial binder was used to formulate CB_0 and CB_1 paints. MB, SA40 and SA50 binders were used to formulate P_MB, P_SA40 and P_SA50, respectively.

**Table 2:** Liquid water uptake values after 14 days of immersion in water or 3.5 wt% NaCl solution of the film cast at 23 °C (MB, SA40, SA50 and CB) and at 60 °C (SA40_T60 and SA50_T60).

	Liquid Water Uptake (Weight Gain %/14 Days)					
	MB	SA40	SA40_T60	SA50	SA50_T60	CB
H_2_O	18.1	22.9	14.7	24.9	12.0	20.2
NaCl	7.2	14.8	4.1	17.1	6.6	11.6

**Table 3:** Mechanical stability test of MB, SA40, and SA50 latexes.

		MB (ppm **)	SA40 (ppm **)	SA50 (ppm **)
pH	7	330	219	290
	8 *	30	48	86

* pH correction made by addition of NH_4_OH solution. ** ppm of coagulum after filtration with 325 mesh filter.

**Table 4:** Adhesion test results of the formulated paints on steel. According to the ASTM D3359 [43] the adhesion performance is defined in a scale from 1B (the lowest) to 5B (the highest).

Sample		P_MB	P_SA40	P_SA50	P_SA40_T60	P_SA50_T60	CB_1	CB_0
Adhesion	A	1B	4B	4B	4B	4B	4B	2B
	U	1B	4B	4B	5B	5B	5B	4B

## Data Availability

The data presented in this study are available on request from the corresponding author.

## Supplementary Materials

The following supporting information can be downloaded at: https://www.mdpi.com/article/10.3390/polym14020316/s1, Figure S1: TEM micrographs of SA40 and of SA50 latex particles, Figure S2: TEM micrographs of the cross section of films cast from SA40 and of SA50 latex at room temperature, Figure S3: Bode plots of coating based on SA latexes and MB latex after exposure to the salt spray test. Reproduced by permission of ACS, Table S1: MB seed formulation, Table S2: Formulation used to synthetize MB_S and MB_D waterborne binders, Table S3: Batch mini-emulsion polymerization recipes used to prepare the seeds, Table S4: Seeded semi-batch emulsion polymerization recipes used, Table S5: Particle size (dp), the final solids content (SC), the minimum film formation temperature (MFFT), glass transition temperature (Tg), melting temperature (Tm), heat of fusion (∆Hf) and crystallinity (Xc) of the synthesized latexes.
