# Supplementary material for: Taking Advantage of Phosphate Functionalized Waterborne Acrylic Binders to Get Rid of Inhibitors in Direct-to-Metal Paints
Source: Polymers (Basel). 2022 Jan 13;14(2):316. doi: 10.3390/polym14020316 (PMC8778291; doi:10.3390/polym14020316)
Supplement: Supplementary file 1 [file polymers-14-00316-s001.zip › polymers-1494218-supplementary.pdf]

Supplementary Material for Article:

Article

Taking Advantage of Phosphate Functionalized Waterborne Acrylic Binders to Get Rid of Inhibitors in Direct-To-Metal Paints

Stefano Chimenti [1], Marco Cerra [2], Tito Zanetta [2], Jose Ramon Leiza [1,*] and María Paulis, [1,*]

[1] POLYMAT, Kimika Aplikatua Saila, Kimika Fakultatea, University of the Basque Country UPV/EHU,
Joxe Mari Korta Zentroa, Tolosa Hiribidea 72, 20018 Donostia-San Sebastián, Spain; stefano.chimenti@polymat.eu

[2] Vinavil SPA, Via Toce, 7, 28844 Villadossola, Italy; m.cerra@vinavil.it (M.C.); t.zanetta@vinavil.it (T.Z.)

* Correspondence: jrleiza@ehu.eus (J.R.L.); maria.paulis@ehu.eus (M.P.)

S1. Synthesis of the phosphated waterborne polymeric dispersion

Tables S1 and S2 report the recipes for the synthesis of the seed of poly(MMA-co-BA) and the final waterborne latexes of the same composition with a total of 50% solids content and phosphates functionalities, respectively.

Table S1. MB seed formulation

Reagent	*Initial load* (g)	*Stream* (g)
MMA	-	31.3
BA	-	31.3
Water	436.7	-
DOW	0.630	-
KPS	0.313	-
$NaHCO_3$	1.035	-

Table S2. Formulation used to synthetize MB_S and MB_D waterborne binders.

LATEX	MB	MMA(g)	BA(g)	KPS(g)	SIP(g)	Water(g)

	(seed)(g)					
MB_S	107	119	119	0.313	5	149.77

Tables S3 and S4 report the recipes for the synthesis of the seed of poly(SA) and the final waterborne latexes based on Poly(SA/MMA-co-BA) with a total of 50% solids content and phosphates functionalities, respectively.

Table S3. Batch mini-emulsion polymerization recipes used to prepare the seeds.

**SEED**		***Mini-emulsion* (g)**	
		**SA40s**	**SA50s**
Oil phase	SA	90	112.5
Water phase	DOW	1.8	2.25
	WATER	247.5	247.5
	$NaHCO_3$	0.144	0.144
Initiator shot	AIBN	0.45	0.56

Table S4. Seeded semi-batch emulsion polymerization recipes used.

**LATEX**	**Seed (g)**	**MMA (g)**	**BA (g)**	**KPS (g)**	**SIP (g)**	**Water (g)**
**SA40**	339.9	67.5	67.5	0.675	4.5	27.5
**SA50**	362.9	56.2	56.2	0.560	4.5	27.5

*Poly(MMA-co-BA) with 13 % solids and with 1 wbm % of DOW

S2. Paint formulation ingredients

Waterborne paints are constituted by a large amount of raw of materials. Some of them are briefly described here.

The pigment is the discontinuous phase giving additional or improved properties to the coatings. These are solid particles insoluble in the application medium, and although most pigments are natural minerals, some are synthesized. Pigments can be divided into two different categories: functional or extenders. Functional fillers, such as titanium dioxide and precipitated calcium carbonate, improve or provide film properties whereas extenders, such as clay, chalk, and ground calcium carbonate

(GCC) are cheaper and they are primarily used to increase the volume of a given coating (and therefore to lower the formulation costs of the final product).

Among the additives, the thickeners are very important in order to achieve the optimum rheology profile desirable for the final applications. If the rheology of the paint is not the proper one, it can affect the paint in terms of manufacturing, storage and application. Paints based on binders that are dissolved in organic solvents exhibit a rheological profile that is very favorable with respect to application and film formation. However, when a water-based binder dispersion is mixed with a pigment dispersion, a completely different rheological profile is obtained. For such systems the viscosity over the whole shear rate is usually too low. This disadvantage makes the use of rheology modifiers necessary for water-based dispersion paints.

During the production of waterborne paints, pigments particles have to be dispersed in water at high speed. In this dispersing process, due to the high speed, pigments agglomerates are dispersed to primary particles. However, in the absence of a dispersing agent these primary particles can flocculate again. The flocculation is due to the Brownian motion of the particles. Small particles move randomly in a suspension, collide with each other and flocculate, reducing their total surface energy. Therefore, when formulating a coating, the role of the dispersing agent is very important, to ensure well dispersed pigmented coatings with good final performances. If pigment particle aggregates are present, final properties such as gloss, opacity, strength, color distribution, and storage stability will be highly affected.

Foam is created when air is introduced into paint during manufacturing or application. Complex paint formulations include several vehicles that promote foam stabilization (i.e., wetting agents, dispersants, and emulsifiers). These surfactants stabilize the foam due to one or several mechanisms available to them including hydrogen bonding, ionic, and van der Waals forces. Foaming is highly undesirable and almost unavoidable. It reduces manufacturing efficiency and causes film defects. This necessitates the use of foam control agents—surface-active additives that prevent, reduce, or eliminate foaming during manufacturing and application.

Water-based paints are prone to contamination and spoilage by bacteria and occasionally unicellular fungi (i.e., yeast) and filamentous fungi. Bacterial growth requirements include water, a carbon source, an energy source, oxygen for aerobes, and other macroelements such as nitrogen and phosphorus. The majority of bacteria grow between 10 and 50 °C and pH range of 3–11. Paint formulations provide an adequate supply of the minimal nutrients at the ideal pH and temperature to maintain bacterial growth. To prevent these problems from occurring, the appropriate biocide must be incorporated into the coating formulation. In addition to being cost-effective, the biocide should be compatible with all coating components, be stable within a range of pH, temperature, and time period, have no effect on rheology, impart no discoloration or odors to the coatings, be water soluble in concentrations used, and be environmentally acceptable

S3. Properties of the synthesized phosphated latexes

Some of the main properties of the synthesized latexes are reported below, namely the particles size (dp), the final solids content, the minimum film formation temperature, and their thermal properties.

Table S5. Particle size (dp), the final solids content (SC), the minimum film formation temperature (MFFT), glass transition temperature (Tg), melting temperature (Tm), heat of fusion (ΔH_f) and crystallinity (X_c) of the synthesized latexes.

Reagent	Dp (nm)	SC (%)	MFFT (°C)	Tg (°C)	Tm (°C)	ΔH_f (j/g)	X_c (%) *
MB	123	50	9	14.7	-	-	
SA40	180	45	10	14.5	50.3	37.7	17.1
SA50	176	45	10	15.1	50.2	45.6	20.7

* Calculated considering ΔH_f^0 = 219.5 j/g [1]

From the DSC analysis we, determined a melting point for the latexes containing crystalline nanodomains based on stearyl acrylate. These crystalline domains were also observed in the transmission electron microscopy analysis (TEM). In Figure S1 a clear core-shell morphology can be observed in which the crystalline core more electron dense resulted darker in comparison to the amorphous shell that belongs to the (meth) acrylates moieties.

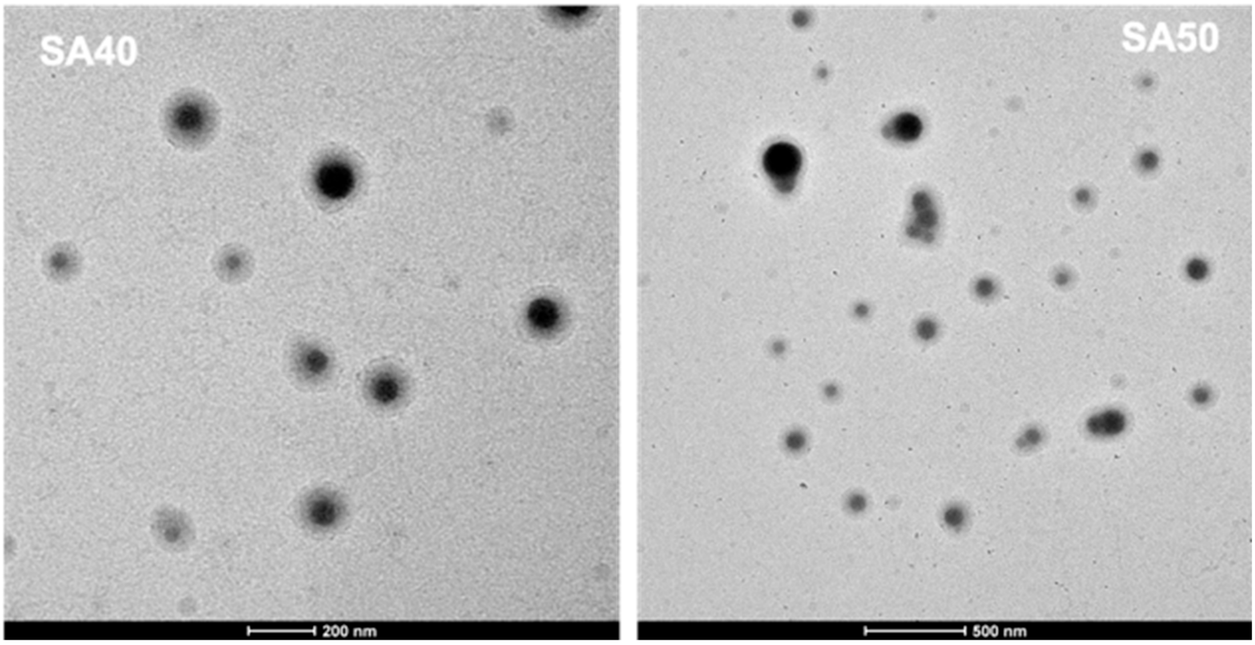

Figure S1. TEM micrographs of SA40 and of SA50 latex particles

TEM micrographs (figure S2) of the cross sectioned films cast from SA latexes show the homogeneous dispersion of the crystalline nanodomains into the polymeric matrix by the presence of well-defined dark zones that represent the electron dense poly(sterаryl acrylate) crystals.

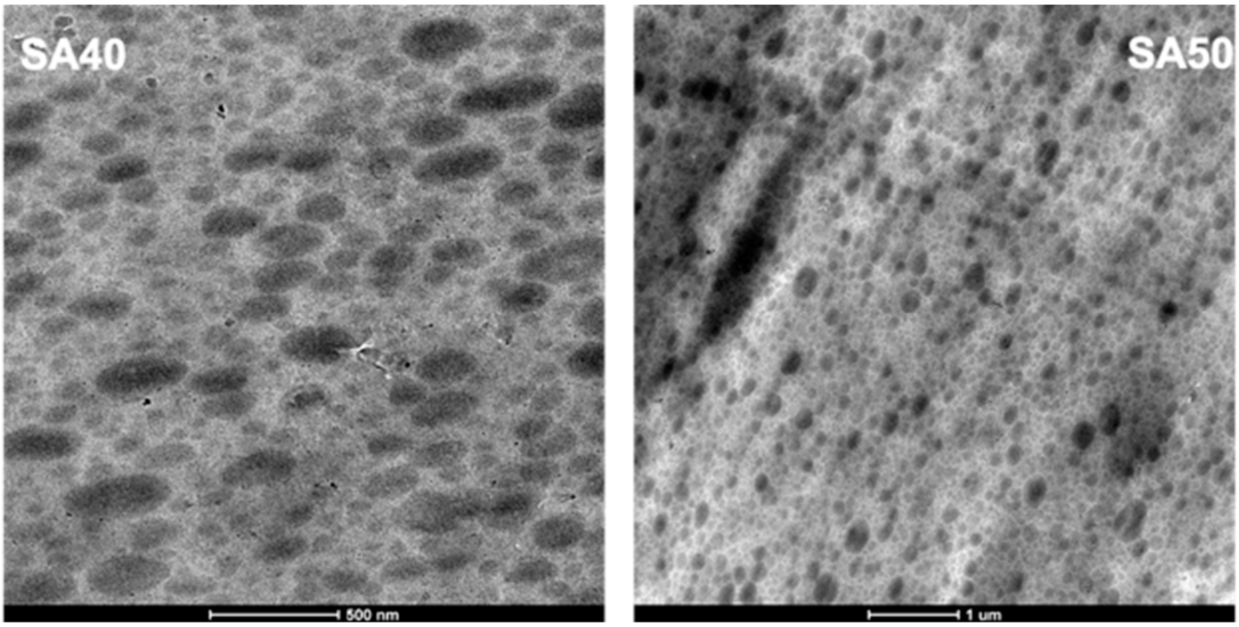

Figure S2. TEM micrographs of the cross section of films cast from SA40 and of SA50 latex at room temperature.

S4. Anticorrosion properties of the synthesized latexes

Figure S3 reports the Bode plots of the film cast from SA latexes applied on a steel substrate collected after the exposure of the coated steel under harsh corrosive conditions produced in a salt spray test[2]. Steel panels coated with the mentioned latexes were exposed up to 1000 h to a 5 wt% NaCl fog at 35 °C and every 200 h the corrosion resistance of the coating was assessed by electrochemical impedance spectroscopy (EIS) and then represented by means of the Bode plot.

As it can be seen, films cast from SA latexes dried at 60 °C provided corrosion protection to the steel for 800 h of exposure in the salt spray test whereas the coating based on MB latex provide protection for 400 h.

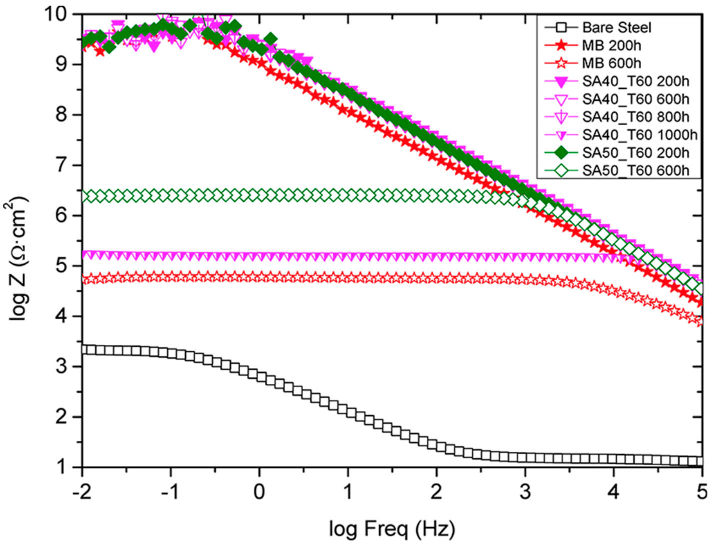

Figure S3. Bode plots of coating based on SA latexes and MB latex after exposure to the salt spray test. Reproduced by permission of ACS.

REFERENCES

1. Jordan, E. F.; Feldeisen, D. W.; Wrigley, A. N., Side-chain crystallinity. I. Heats of fusion and melting transitions on selected homopolymers having long side chains. *Journal of Polymer Science Part A-1: Polymer Chemistry* **1971,** *9* (7), 1835-1851.
2. Chimenti, S.; Vega, J. M.; Lecina, E. G.; Grande, H.-J.; Paulis, M.; Leiza, J. R., Combined Effect of Crystalline Nanodomains and in Situ Phosphatization on the Anticorrosion Properties of Waterborne Composite Latex Films. *Industrial & Engineering Chemistry Research* **2019,** *58* (46), 21022-21030.
